# Excitation-dependent fluorescence from atomic/molecular layer deposited sodium-uracil thin films

**DOI:** 10.1038/s41598-017-07456-6

**Published:** 2017-08-01

**Authors:** Ville Pale, Zivile Giedraityte, Xi Chen, Olga Lopez-Acevedo, Ilkka Tittonen, Maarit Karppinen

**Affiliations:** 1 Department of Electronics and Nanoengineering, Aalto University, FI-00076 Aalto, Finland; 2 Department of Chemistry and Materials Science, Aalto University, FI-00076 Aalto, Finland; 3 COMP Centre of Excellence in Computational Nanoscience, Department of Applied Physics, Aalto University, FI-00076 Aalto, Finland; 4 Departamento de Ciencias Básicas, Universidad de Medellín, Carrera 87 # 30-65, Medellín, Colombia

## Abstract

Atomic/molecular layer deposition (ALD/MLD) offers unique possibilities in the fabrication of inorganic-organic thin films with novel functionalities. Especially, incorporating nucleobases in the thin-film structures could open new avenues in the development of bio-electronic and photonic devices. Here we report an intense blue and widely excitation-dependent fluorescence in the visible region for ALD/MLD fabricated sodium-uracil thin films, where the crystalline network is formed from hydrogen-bonded uracil molecules linked via Na atoms. The excitation-dependent fluorescence is caused by the red-edge excitation shift (REES) effect taking place in the red-edge of the absorption spectrum, where the spectral relaxation occurs in continuous manner as demonstrated by the time-resolved measurements.

## Introduction

During the last decade, there has been a growing interest in developing new biomaterials for bioelectronics and photonic applications^1, 2^; the driving motivation is to utilize ecologically safe and environmentally sustainable, cheap and renewable material sources for frontier applications^3^. Another excitement arises from the diversity of exceptional functional properties created by nature for many biological materials^4, 5^. As the most notable material candidate, deoxyribonucleic acid (DNA) has been investigated for various bio-related electronic and photonic applications; it is a renewable resource and can be obtained from various waste and natural sources, such as salmon sperm^6^ and vegetation^7^. Moreover, DNA has excellent optical properties and it has been utilized in polymer waveguides^8^, nonlinear optical devices^9^ and DNA-templated photonic arrays^10^. In optoelectronic applications DNA has been employed as an electron transport layer (ETL) in organic light emitting diodes (OLEDs) and as a gate dielectric in organic field emitting transistors (OFETs)^11, 12^.

Compared to the massive interest in DNA, its constituent parts, *i.e*., the nucleobases (NBs), have received considerably less attention as potential building blocks for bioelectronic material applications. This is surprising, as their rich chemical structures can facilitate aromatic stacking and different hydrogen-bonding schemes to create three-dimensional supramolecular assemblies, like in the natural systems^13–15^. The smaller size of the NB molecules compared to DNA is a clear advantage when considering their employment as precursors in vacuum-based thin-film deposition techniques^16, 17^. Another advantage concerns the better controllability of the desired functionality, as the properties are defined more precisely for the monomeric NBs than for the monumental DNA polymers with only the approximate molecular masses known. Moreover, NBs can be obtained both synthetically and from natural sources^18, 19^ making them a cheap and abundant material resource.

The NBs are electrical insulators with fairly large HOMO-LUMO band gaps (3.6–4.1 eV)^4^, and unfortunately typically exhibit exceedingly low fluorescence quantum yields^20, 21^. For example, the quantum yield of pyrimidines in water is only 0.01^22^. However, it has been reported that subtle modifications to the NB conjugated system could lead to a substantial fluorescence quantum yield increases, opening up novel possibilities in radiative pathway engineering^23, 24^.

**Figure 1:**
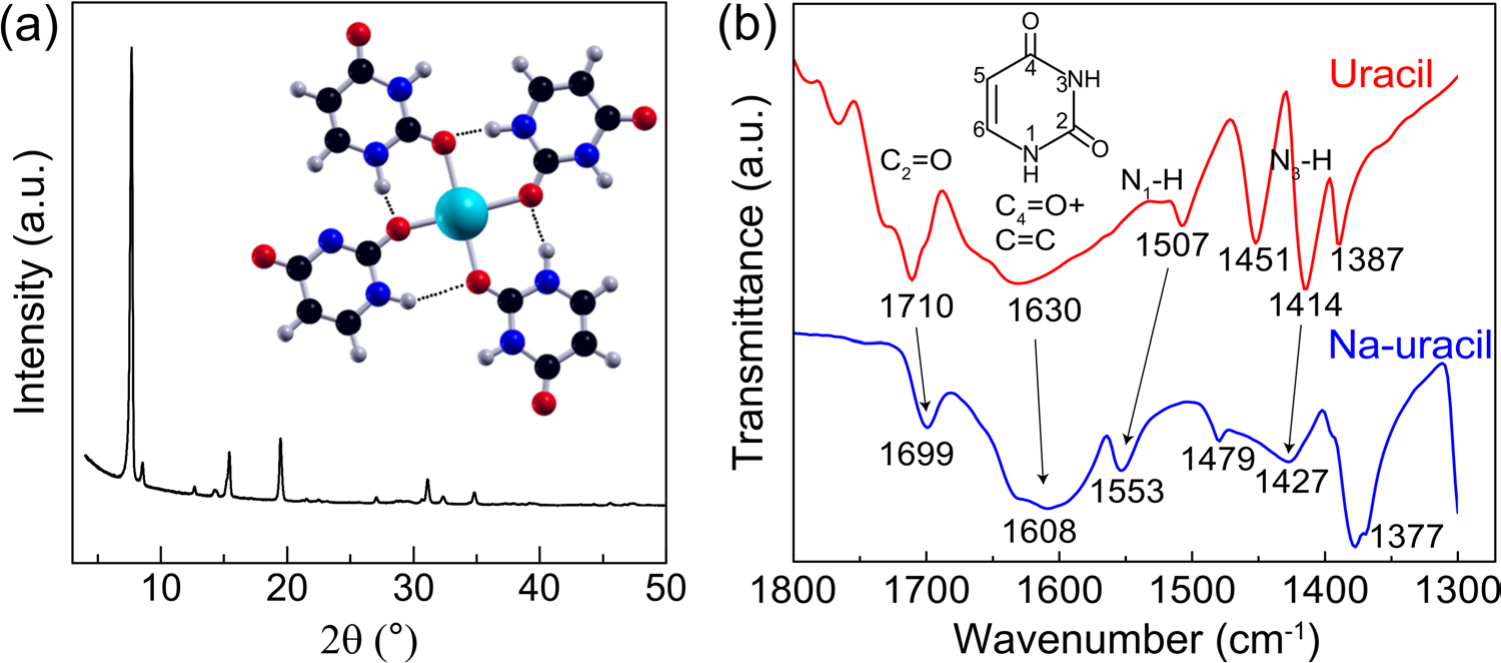
GIXRD pattern (**a**) and FTIR spectrum (**b**) recorded for our Na-uracil thin films (in the latter case uracil spectrum is also shown for reference); the tetrametric structure and the numbering of the atoms in uracil molecule are shown in the insets, respectively.

In our recent work, we demonstrated the possibility to build crystalline 3D networks in which the NB molecules are linked together via sodium atoms using the strongly emerging atomic/molecular layer deposition (ALD/MLD) thin-film technique;^25^ our proof-of-the-concept data were for uracil as the NB constituent^26^. The ALD/MLD technique offers simple but powerful routes to fabricate novel inorganic-organic functional thin-film materials even on structurally most demanding substrates such as flexible, porous and nanostructured surfaces, with atomic-level thickness control; for a recent review of the technique and examples of crystalline hybrid thin films fabricated thereof, see refs 25, 27–29. In the case of the ALD/MLD realized Na-uracil assemblies, our thorough experimental and computational characterization gave evidence that the uracil molecules in these hybrid networks are in a planar tetrametric configuration (see the inset in Fig. 1a). Moreover, the assemblies showed a substantially red-shifted fluorescence between the two pump wavelengths of 280 and 400 nm. This phenomenon is quite rare in conventional fluorophores, such as organic dyes or inorganic quantum dots^30^. Typically, the emission takes place from the lowest excited state of given multiplicity regardless of the excitation wavelength, which is also known as the Kasha’s rule^30–33^.

The shifting behavior of the fluorescence emission induced by the changing excitation wavelength in the red edge of the absorption spectrum is also referred to as the red-edge excitation shift (REES)^30, 34^. This effect was originally observed in rigid and viscous media, such as low-temperature glasses and polymer matrices that display only modest excitation-dependent shifts^34^. Furthermore, more recently REES has been observed in self-assembled peptide nucleic acids, in reduced graphene oxide and decameric monoacylglycerol nanoparticles^35–38^. These red-edge effects are assumed to result from the distribution of interaction energies between the excited-state fluorophore and the molecular environments, as well as the slowed relaxation/solvation dynamics of the molecular environments.

In this work, we report the results of a detailed spectroscopic analysis for our Na-uracil thin film samples. Absorption measurements and simulations indicate that the assemblies give rise to broadening and long spectral tail on the red edge of the absorption spectrum. Steady-state fluorescence measurements illustrate a wide spectral range of excitation-dependent fluorescence in the visible wavelength range. Furthermore, the results revealed from the time-resolved measurements are in line with a continuous spectral relaxation model, or the REES phenomenon. Thus, with such intriguing optical properties combined with the uniquely attractive way of their synthesis the present Na-uracil thin films and the readily imaginable other similar nucleobase-derived hybrid materials could open new pathways for bioinspired optoelectronic and photonic applications.

## Results and Discussion

Our Na-uracil thin films were grown in an atomic/molecular layer-by-layer fashion through ligand-exchange reactions from Na(thd) and uracil precursors in ALD reactor at 300 °C. 200 ALD/MLD cycles yielded visually homogeneous and stable hybrid Na-uracil thin films with a thickness of 95 nm. A schematic presentation of the structure is shown in Fig. 1, along with a typical Fourier-transform infrared (FTIR) spectrum and a grazing-incidence x-ray diffraction (GIXRD) pattern recorded for the films to evidence, respectively, the presence of the targeted organic moieties and the crystallinity of the films. A more detailed description of the sample preparation and characterization is found in ref. 26, and in Methods.

In our previous work^26^, we demonstrated hydrogen bond interactions between the protons of the N1-H and N3-H groups and the oxygen atoms of carbonyl groups that act as proton donors and hydrogen acceptor sites, respectively. Upon deprotonation, the C=O bond is affected because of the delocalization of the *π*-electrons in the heterocyclic ring. In Fig. 1b, the shifts toward the lower frequencies from 1710 to 1699 cm^−1^, and from 1630 to 1608 cm^−1^, are assigned to the binding of the sodium ions through oxygen^39, 40^. The N-H resonance at 1414 cm^−1^ shifts to 1427 cm^−1^, which is due to the N3-H hydrogen bond coupling with C=O. Moreover, the blue-shifted N1-H resonance from 1507 to 1553 cm^−1^ indicates of N1-H…O hydrogen bond coupling^41^. These shifts (cf. Figure 1b) indicate that C=O and N-H are involved in strong hydrogen bonds.

**Figure 2:**
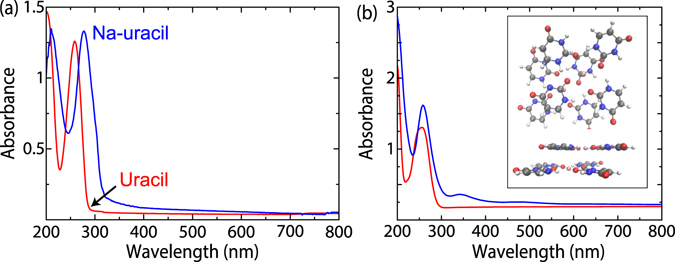
(**a**) The measured absorption spectra of uracil reference (red) and Na-uracil thin film (blue). (**b**) Calculated absorption spectra of uracil (red) and Na-uracil (blue) and the Na-uracil structure used for modeling in the inset. The individual optical transitions have been folded into smooth curves by using a Gaussian width of 0.2 eV.

For the optical characterization, we measured UV-vis absorption for a broad spectral range of 200–800 nm for both our uracil reference and Na-uracil thin film deposited on quartz substrates, see Fig. 2a. For the uracil reference, the characteristic absorption peaks due to the *n*/*π* ^*^ and *π*/*π* ^*^ transitions are seen at 200 nm and 260 nm, respectively^21^. For our Na-uracil sample, both peaks are substantially red-shifted. In comparison to the uracil reference, the *π*/*π* ^*^ peak shifts from 258 to 277 nm and the FWMH is broadened from 34.5 to 46.0 nm. Moreover, the Na-uracil sample exhibits a long spectral trail extending towards the visible wavelength range.

To gain more insight into the shifting and broadening phenomena, we calculated the optical absorption spectra of uracil and Na-uracil by the time-propagation Time Dependent Density Functional Theory (TDDFT) with linear combination of atomic orbitals, implemented in the GPAW code^42–44^. Based on the previous work^26^, we used eight uracil molecules and two Na atoms in two layers to model the Na-uracil network, as shown in Fig. 2b. The overall Na-uracil unit cell is neutral as it contains three neutral uracils, Na-cation and one deprotonated uracil. In the calculation, the weak delta pulse perturbation was applied in 3 spatial directions then the spectrum was averaged. The system was propagated for 4000 steps using a 5 atto second time-step, which corresponds to a total simulation time of 20 fs. The double-zeta plus polarization (DZP) basis sets and B3LYP hybrid exchange-correlation functionals were used in all the calculations^45, 46^. Since the dipole moment is generated by displacements in the charge density, the peaks in the optical absorption spectrum signify nearly harmonic oscillations herein. Therefore, we calculated the Fourier transforms of time-evolution of the density at the resonant frequencies to analyze the peaks in the optical spectra, called absorption-induced density in this work.

The calculated optical spectra agree well with the measured ones. The absorption peak of Na-uracil (FWHM = 50.9 nm) shifts to lower energy and becomes broader as shown in Fig. 2b in comparison to the uracil spectrum (FWHM = 46.2 nm), which is in accordance with the measured spectra. In order to characterize the peaks, we calculated the absorption induced density, shown in Figure S1. The peak in the calculated spectrum of uracil near 248 nm, and the peak in the calculated spectrum of Na-uracil near 265 nm both originate from *π*/*π* ^*^ transitions. Although sodium and planar hydrogen bonds are important for forming the ordered Na-uracil structure, they do not directly contribute to the transitions here. As shown in Figure S2, the four LUMO states of the Na-uracil correspond to the LUMO state of the uracil molecule, but these states are localized in different uracils and spread over an energy range of 0.42 eV, which could be responsible for the shifting and broadening of the absorption peak of Na-uracil thin film. The experimental long spectral tail can be obtained from the simulated low and broad energy excitation peaks at 350 and 470 nm. The induced density changes in these low energy excitations are not localised but completely delocalised in the 8 uracil (two layer) model system. Increasing the size of the model system could further broaden these low energy peaks^47^.

**Figure 3:**
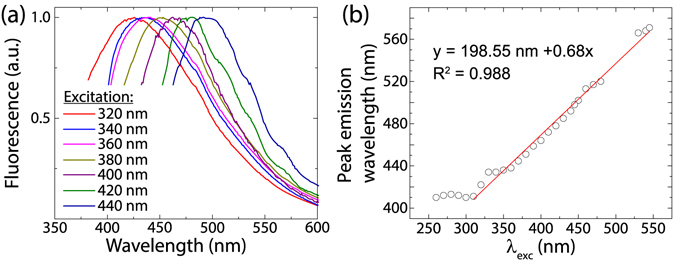
Illustration of the broad excitation-dependent fluorescence of our Na-uracil thin-film samples: (**a**) normalized emission spectra recorded with various excitation wavelengths, and (**b**) the essentially linear dependency of the emission wavelength on the excitation wavelength.

When measuring the steady-fluorescence spectra, the samples exhibited strong and bright fluorescence signals. The strongest emission was detected in the blue region with the 280 nm pump wavelength. In addition, the samples showed an excitation-dependent fluorescence emission in a broad wavelength range that starts taking place after the excitation wavelength of 310 nm. The excitation-dependent fluorescence is illustrated in Fig. 3a that presents the normalized fluorescence spectra, where the peak emission wavelength shifts from 411 to 492 nm for excitation wavelengths ranging from 310 to 440 nm, respectively. As shown in Fig. S3a, the fluorescence intensity decreases with a constant spectral shape. This decrease is essentially linear as demonstrated in Figure S3b; when excited at 440 nm, the emission intensity is lower by 90% when compared to the fluorescence obtained with the 330 nm excitation wavelength. To investigate the emission-excitation dependency in more detail, we plotted the emission peak wavelengths as a function of the excitation wavelength shown in Fig. 3b. The data demonstrate a good linear dependency (R^2^ = 0.988) with a slope of 0.68 between the emission and excitation wavelengths. The slope, defined as the change in the peak emission wavelength for a change in the excitation wavelength, *i.e*., ${\rm{\Delta }}{\lambda }_{{\rm{e}}{\rm{m}}}/{\rm{\Delta }}{\lambda }_{{\rm{e}}{\rm{x}}{\rm{c}}}$, stays essentially constant up to 540 nm. The fluorescence at 540 nm excitation is relatively weak, but still distinguishable (cf. Fig. S4).

Next, we carried out time-resolved fluorescence measurements to elucidate the excited state dynamics and to shed light whether REES could be behind the observed broad excitation-dependent emission. Usually, the excited state effects are investigated by measuring the time-resolved emission spectra (TRES), which illustrates how the emission spectrum evolves as a function of time after a pulsed excitation. Moreover in terms of the REES model, one should observe continuous spectral shifts with time by the TRES analysis^30^. The analysis was started by measuring the fluorescence emission decays along the whole emission spectrum for the 400 nm excitation, where the lifetimes from the time-resolved decays were analyzed as described previously using a three exponential model^48^. A more detailed description of the TRES calculation is presented in the Supplementary.

**Figure 4:**
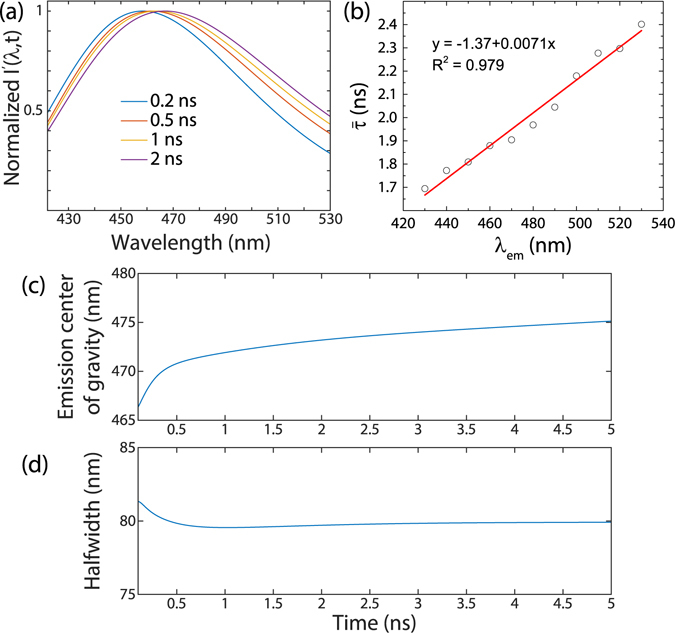
Results of TRES and lifetime analysis. (**a**) TRES spectra taken at different instances of time after the excitation, (**b**) Average lifetime as the function of emission spectrum wavelength, (**c**) Emission center of gravity as a function of time after excitation and (**c**) the fluorescence emission halfwidth as the function of time after excitation.

Figure 4a shows the normalized TRES spectra as a function of time following the moment of excitation. Distinctively, the data clearly demonstrate the shifting of the fluorescence to longer wavelengths as time progresses, which is consistent with the REES model. The REES model also predicts that the excited species that are excited to the Franck-Condon (FC) state (*i.e*., unrelaxed state) should emit their light at higher energies than the relaxed (R) state. In addition, since the relaxation process from the FC state to the R state has a specific time constant, the emission observed at longer wavelengths should have longer average lifetimes. Actually, this is exactly what we observe in Fig. 4b, which presents the intensity-weighted average lifetime as a function of emission spectrum wavelength. The measured data exhibit a linear increase in the average lifetime from 1.7 to 2.4 ns. This demonstrates that the species in the FC state emit their light earlier than the species in the R state and that the transition between the FC and R states happens in a continuous manner.

In addition, we investigated the evolution of the fluorescence center of gravity and the emission spectra half width as a function of time, presented in Fig. 4c and Fig. 4d, respectively. These graphs were calculated using equations (S1) and (S2). The fluorescence center of gravity displays a dynamic Stokes shift, where the majority of the changes occur 1 ns after the excitation moment. After this, the emission center of gravity keeps slowly shifting towards longer wavelengths. The half width in Fig. 4d stays constant during the spectral relaxation, which is also corroborated by the relatively static spectral shape of fluorescence as shown in Fig. 4a. These observations are consistent with continuous spectral relaxation provided by the REES model.

The spectroscopic characteristics observed in the steady-state and time-resolved fluorescence measurements imply a model, where the spectral relaxation takes place in a continuous manner. This kind of behaviour is especially pronounced in systems, where the solvation dynamics are slowed down to the same order of magnitude as the fluorescence lifetime of the excited moiety^34^. We suspect that in our samples, the extensive hydrogen-bonding network between the uracil molecules could form such a motionally restricted media to enable favorable conditions for REES^38^. The continuous relaxation model is supported by the TRES analysis that shows a gradual shift toward longer wavelengths with a constant spectral shape and FWHM. In addition, the increase of the mean lifetime with increasing emission wavelength is a characteristic fingerprint of the continuous spectral relaxation model and REES^30^.

For our Na-uracil films the peak emission wavelength changes from 410 to 570 nm as the excitation wavelength is changed from 410 to 540 nm, which corresponds to an approximately 150 nm emission shift. This giant emission red-shift and the ratio of ${\rm{\Delta }}{\lambda }_{{\rm{em}}}/{\rm{\Delta }}{\lambda }_{{\rm{exc}}}=0.68$ reflects the slow solvation dynamics in the Na-uracil assembly. Typically, the reported REES effects are quite modest; 10–40 nm emission shifts have been observed for fluorophores in vitrified or viscous materials, such as ionic liquids, polymers and proteins^49–51^. Moreover, the lack of exponential saturation of ${\rm{\Delta }}{\lambda }_{{\rm{em}}}/{\rm{\Delta }}{\lambda }_{{\rm{exc}}}$ in Fig. 3b indicates that the completely solvated state is not reached during the fluorescence lifetime.

## Conclusions

We have revealed exciting optical properties for a new type of inorganic-organic 3D structure incorporating nucleobase molecules. Our Na-uracil material exhibits an intense blue and excitation-dependent fluorescence in the visible wavelength range. The observed excitation-dependent fluorescence and dynamic Stokes shift by themselves are rare phenomena in organic molecular systems. Moreover, the wide spectral range of the excitation-dependent fluorescence in sodium-uracil films could be extremely beneficial for organic optical devices.

The underlying mechanism could be identified as a so-called REES effect previously observed for some rare cases; the required fact that the spectral relaxation takes place in a continuous manner was shown by the time-resolved measurements. The REES effect can open new possibilities in photonic applications as the emission wavelength can be simply changed by choosing a different excitation wavelength. We expect that our approach could pave the way for new bioinspired hybrid materials with nucleobases for optoelectronic and photonic applications.

## Methods

We performed the ALD/MLD deposition of the Na-uracil thin films on diced (3.5 × 3.5 cm^2^) silicon (100) and on quartz substrates in a commercial ALD reactor (F-120 by ASM Microchemistry Ltd). The process was done at 300 °C and nitrogen (99.999%; Schmidilin UHPN 3000 N_2_ generator) was used as a carrier and purging gas. The pressure was kept in 2-4 mbar during the film deposition. The films were grown from in-house made Na(thd) (thd: 2,2,6,6-tetramethyl-3,5-heptadionate)^52^ and commercial uracil (Sigma Aldrich) solid precursors. The precursor powders were kept in glass crucibles inside the reactor, at 250 °C and 235 °C, respectively, for sublimation. The ALD/MLD pulsing sequence was: *n*[Na(thd) 1.5 s → N_2_ purge 2 s → uracil 2 s → N_2_ purge 4 s], which was repeated for *n* = 200 cycles.

The GIXRD spectra were collected using PanAnalytical X’Pert MPD Pro Alfa X-ray diffractometer using CuK_*α*−1_ radiation. The FTIR spectra were collected with Nicolet 380 (ThermoFisher Scientific) spectrometer mode with 1 cm^−1^ resolution and averaging of 64 scans. The film thickness was measured using TopoMetrix Explorer atomic force microscope (AFM). The absorbance was measured using a Lambda 950 (Perkin-Elmer) UV-vis spectrometer using an integrating sphere. Quantamaster 40 (Photon Technology International) fluorometer was used to record the steady-state fluorescence spectra along with appropriate long pass filters to block the excitation light. The emission and excitation slits used in the measurements were set to 1.25 nm and the fluorescence spectra were corrected by using instruments excitation and emission corrections provided by the manufacturer. The time-resolved measurements were performed using a frequency-doubled, mode-locked Ti:S laser (Coherent, Mira 900-F, rep. rate 76 MHz, pulse width ~200 fs). The fluorescence decay was collected using a Peltier cooled microchannel plate photomultiplier tube along with single photon counting electronics (B&H).

## Electronic supplementary material

Supporting material
