# Supplementary material for: Excitation-dependent fluorescence from atomic/molecular layer deposited sodium-uracil thin films
Source: Sci Rep. 2017 Aug 1;7:6982. doi: 10.1038/s41598-017-07456-6 (PMC5539323; doi:10.1038/s41598-017-07456-6)
Supplement: Supplementary file 1 — Supporting material [file 41598_2017_7456_MOESM1_ESM.pdf]

Supporting Material

Excitation-dependent fluorescence from atomic/molecular layer deposited sodium-uracil thin films

Ville Pale[1,+], Zivile Giedraityte[2,+], Xi Chen[3], Olga Lopez-Acevedo[3,4], Ilkka Tittonen[1], and Maarit Karppinen[2,*]

[1]Department of Electronics and Nanoengineering, Aalto University, FI-00076 Aalto, Finland
[2]Department of Chemistry, Aalto University, FI-00076 Aalto, Finland
[3]COMP Centre of Excellence in Computational Nanoscience, Department of Applied Physics, Aalto University, FI-00076 Aalto, Finland
[4]Department of Basic Sciences, University of Medellin, Carrera 87 # 30-65 Medellìn, Colombia
[+]these authors contributed equally to this work
[*]maarit.karppinen@aalto.fi
KEYWORDS nucleobase, atomic/molecular layer deposition, alkali metal linker, optical properties, red-edge excitation shift

1 Computational results 2

2 Fluorescence spectra with different excitation wavelengths 3

3 Time-resolved emission spectra 4

4 Bibliography 5

1 Computational results

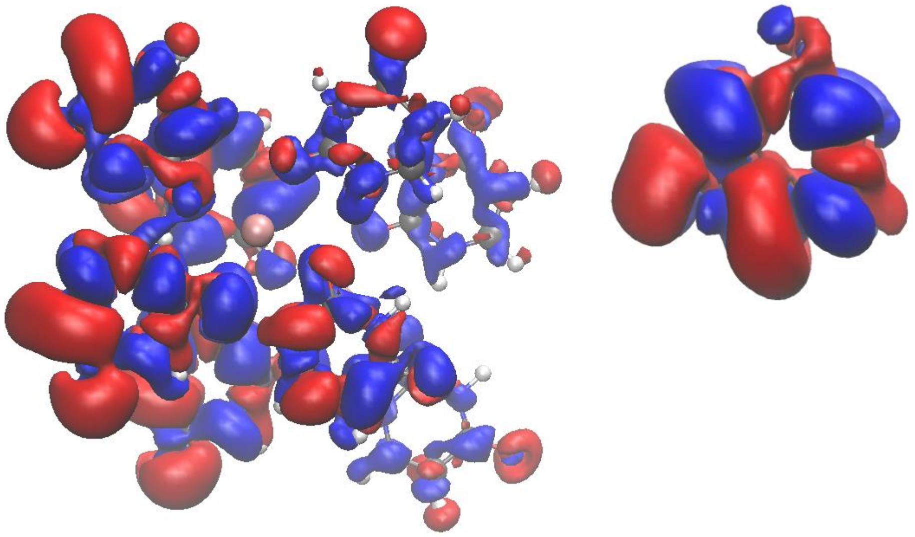

Figure S1. (left) Absorption induced density near 265 nm in Na-uracil (averaged between 248nm-281nm) and (right) absorption induced density near 248 nm in uracil (averaged between 238nm-258nm).

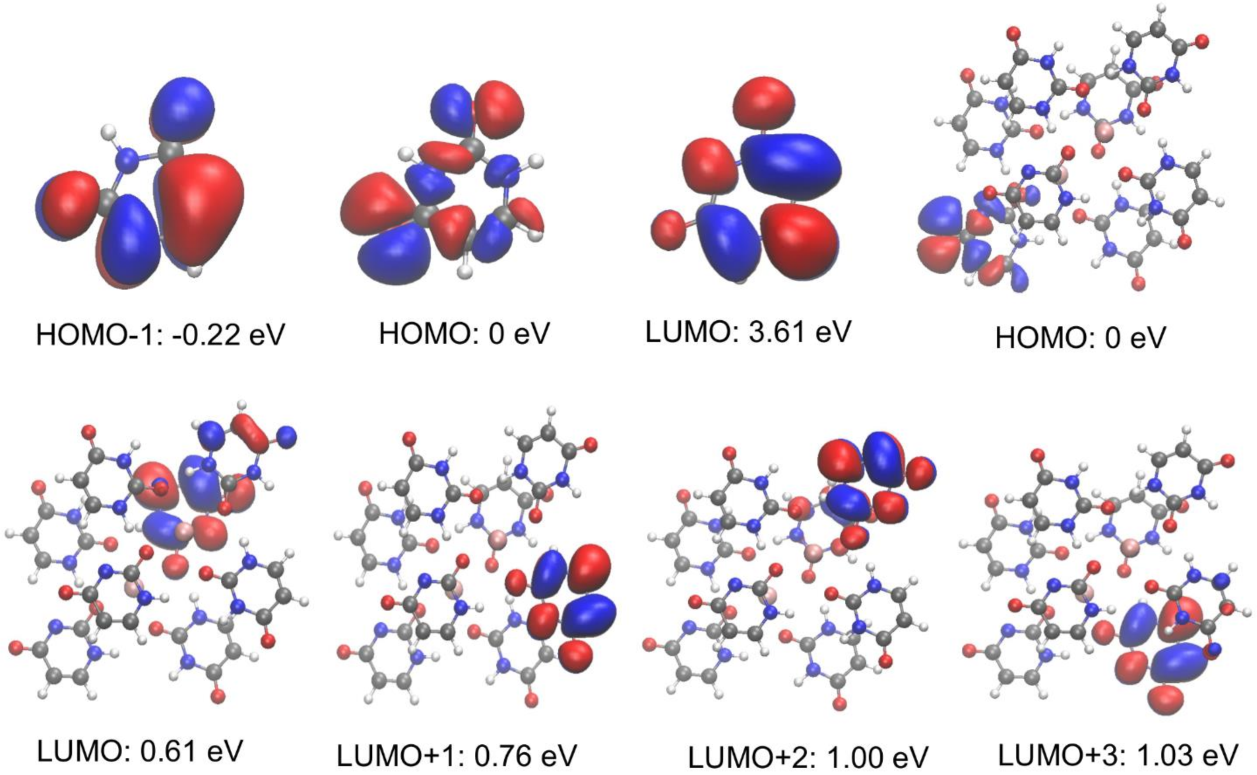

Figure S2. The HOMO-1 to LUMO states of uracil and HOMO to LUMO+3 states of Na-uracil. The numbers given in the figure are the relative energy of the states, while the energy of HOMO is set to 0 eV.

2 Fluorescence spectra with different excitation wavelengths

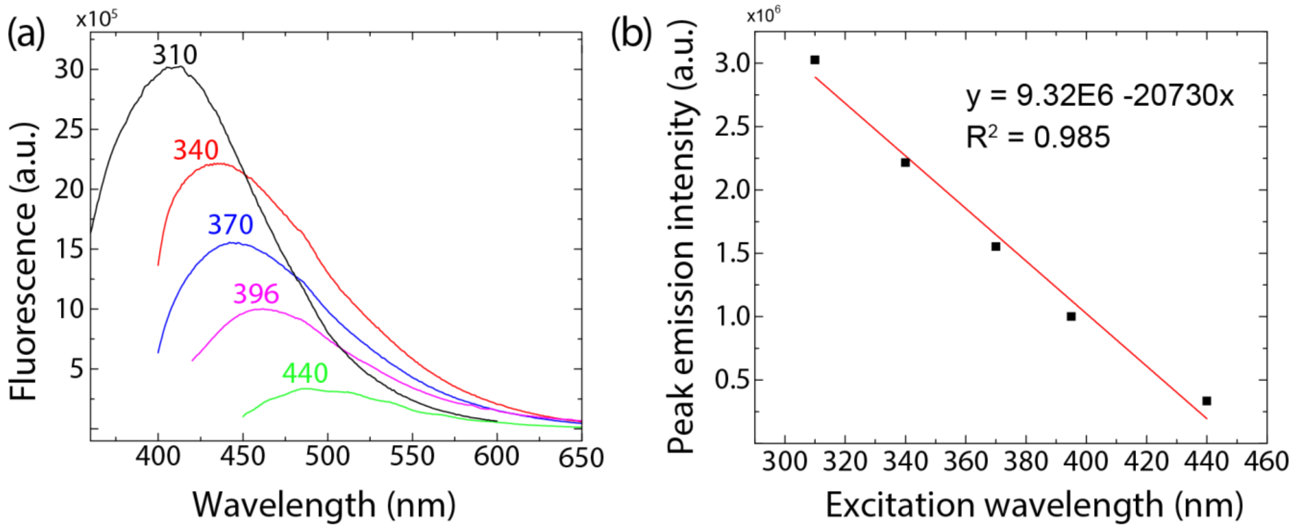

Figure S3. (a) Fluorescence spectra of Na-uracil films with different excitation wavelengths. (b) The peak emission intensity as a function of the excitation wavelength.

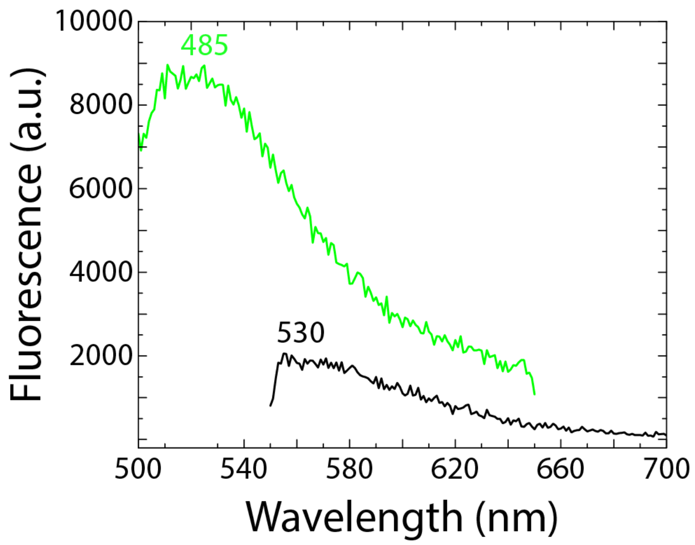

Figure S4.The fluorescence spectra at longer excitation wavelengths.

3 Time-resolved emission spectra

Time-resolved emission spectra (TRES) were constructed from the steady-state fluorescence and from the time-resolved fluorescence decays collected from the entire wavelength range as described previously.[1] The excitation wavelength for TRES measurements was 400 nm and the emission was collected between 420 nm and 530 nm wavelength range with 5 nm spectral resolution using a 409 nm long pass filter. The emission center of gravity was calculated as

$$\lambda_{cg}(t) = \frac{\sum I'(\lambda,t)\lambda}{\sum I'(\lambda,t)} \quad (1)$$

and the time dependent spectral width as

$$\Delta\lambda(t)^2 = \frac{\sum\left(\lambda-\lambda_{cg}(t)\right)^2 I'(\lambda,t)}{\sum I'(\lambda,t)}, \quad (2)$$

where $I'(\lambda,t)$ is the TRES spectra generated from the measured data, also shown in Figure S5. The TRES data was improved by using a log-normal fitting procedure as reported in Ref.[2,3]

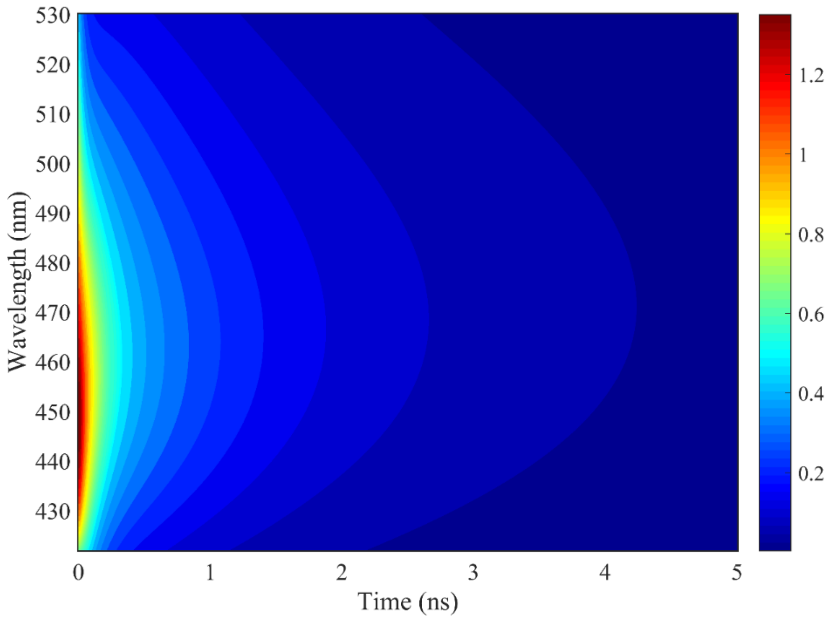

Figure S5. The time resolved emission spectra (TRES) matrix with both the wavelength and time dependency.

4 Bibliography

(1) Lakowicz, J. R. *Principles of fluorescence spectroscopy*, 3rd ed.; Springer: New York, 2006.

(2) Maroncelli, M.; Fleming, G. R. *J. Chem. Phys.* **1987**, *86*, 6221.

(3) Siano, D. B.; Metzler, D. E. *J. Chem. Phys.* **1969**, *51*, 1856.
